# Role of Factor H and Related Proteins in Regulating Complement Activation in the Macula, and Relevance to Age-Related Macular Degeneration

**DOI:** 10.3390/jcm4010018

**Published:** 2014-12-26

**Authors:** Simon J. Clark, Paul N. Bishop

**Affiliations:** 1 Centre for Hearing & Vision Research, Institute of Human Development, AV Hill Building, University of Manchester, Oxford Road, Manchester M13 9PL, UK; E-Mail: Simon.Clark-3@manchester.ac.uk; 2 Centre for Advanced Discovery and Experimental Therapeutics, University of Manchester and Central Manchester University Hospitals NHS Foundation Trust, Manchester Academic Health Science Centre, Manchester M13 9WL, UK; 3 Manchester Royal Eye Hospital, Central Manchester University Hospitals NHS Foundation Trust, Manchester M13 9WH, UK

**Keywords:** age-related macular degeneration, complement factor H, factor H, factor H-like protein 1, factor H related proteins

## Abstract

The recent revolution in age-related macular degeneration (AMD) genetics has demonstrated that genetic alterations affecting the alternative pathway of the complement cascade have a major influence on AMD risk. One of the two most important genetic loci is on chromosome 1 and contains genes encoding complement factor H (FH) and the factor H related proteins (FHR proteins). In macular tissue, especially Bruch’s membrane, relatively high levels of a truncated splice variant of FH called factor H-like protein 1 (FHL-1) are present. Here we discuss how genetic variations may alter the amounts, or by altering their protein sequences, the functions of these proteins. In particular, the common Y402H polymorphism affects the ability of FHL-1 and FH to localize to Bruch’s membrane and the inner choroid because it alters the ability of these complement regulators to bind heparan sulphate (HS) in these structures. In addition, there is an age-related loss of HS from Bruch’s membrane. We hypothesize that a combination of poor binding of the 402H variants of FHL-1 and FH to Bruch’s membrane, combined with a decrease in binding due to age-related HS loss, eventually results in insufficient FHL-1 and FH binding to Bruch’s membrane. This could result in complement activation, inflammation and thereby predispose to AMD.

## 1. Introduction

**Figure 1:**
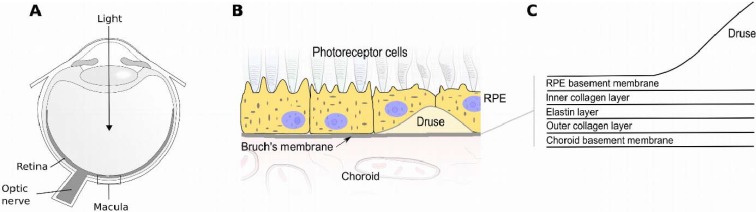
Anatomy of the human eye affected by age-related macula degeneration. (**A**) A cross section of the human eye highlights the macula; (**B**) In early AMD, extracellular deposits called drusen form within Bruch’s membrane underneath the retinal pigment epithelium (RPE); (**C**) Bruch’s membrane is composed of five layers: an elastin core is sandwiched between two collagenous layers and these are surrounded by basement membranes.

Age-related macular degeneration (AMD) is a condition that results in destruction of the macula, which is the central part of the retina (Figure 1). The weight of evidence suggests that the disease process is initiated in anatomical structures underlying the neurosensory retina at the macula including the retinal pigment epithelium (RPE), Bruch’s membrane and choroid. However, ultimately, in late stage AMD the neurosensory retina is destroyed through processes including geographic atrophy (“dry” or atrophic AMD) and choroidal neovascularisation (“wet” or neovascular AMD).

A characteristic finding in early AMD, which precedes the visual loss in late AMD, is the deposition of extracellular material in and around Bruch’s membrane. Bruch’s membrane is a pentilaminar structure containing two basement membranes, two collagenous layers and a central elastin layer (Figure 1). In particular, the clinically characteristic soft drusen are hallmarks of AMD [1]; these yellow deposits form between the inner basement membrane and inner collagenous layer of Bruch’s membrane. They contain, in part, material that is extruded from the adjacent RPE cells and are damaging to RPE function because they disrupt metabolic transport between the RPE and choroid. Drusen also contain a variety of proteins and the discovery of complement components in drusen provided initial evidence for the involvement of the complement system in AMD [2]. Subsequently it emerged that genetics plays a major role in determining AMD risk and many of the genes associated with high and intermediate genetic risk encode components of the alternative complement pathway [3]. Of particular importance are genetic changes in and around the complement factor H (*CFH*) gene on chromosome 1, which encodes factor H (FH), as several of these have been shown to be major modifiers of AMD risk. The other major risk locus is on chromosome 10, in a region containing the *ARMS2*/*HTRA1* genes; however, the mechanisms underpinning risk at this locus are unknown. There are, in addition, a variety of alterations in other genes encoding components of the alternative complement pathway that are associated with AMD including C3, factor B and factor I (FI) [4], highlighting the importance of this pathway in AMD pathogenesis. This review will focus on the role of FH and related proteins.

## 2. Factor H (FH) and the Alternative Complement Pathway

**Figure 2:**
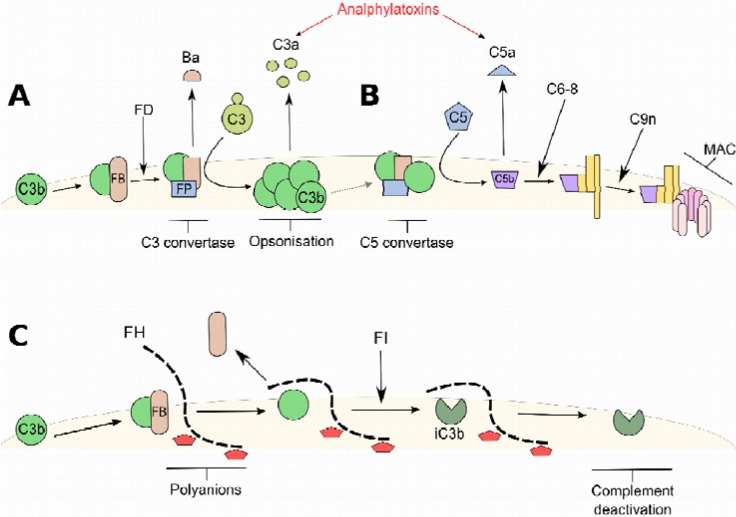
Schematic showing the activation of complement by the alternative pathway. The alternative pathway of complement is constituently active where the protein C3b becomes deposited on all surfaces. (**A**) In the absence of host regulatory proteins, complement factor B (FB) binds the deposited C3b forming a complex that is susceptible to the proteolytic cleavage by factor D (FD). The cleavage of FB bound to C3b creates an active C3 convertase capable of cleaving C3 into C3b; the C3 convertase is stabilized by the binding of factor P (FP, also known as properdin). This is the start of the amplification loop of complement that leads to a rapid and uncontrolled deposition of C3b onto the surface (opsonisation), labeling it for uptake by phagocytosis, accompanied by the release of the potent anaphylatoxin, C3a. This amplification loop feeds into the terminal pathway of complement; (**B**) where the formation of the C5 convertase releases another anaphylatoxin, C5a, and eventually leads to the formation of the membrane attack complex (MAC) that is inserted into the membranes of cells causing lysis and death; (**C**) Complement factor H (FH) derived from the blood, or locally in the eye following secretion by the RPE, is recruited to host surfaces through interactions with polyanions (charged sugar molecules). FH can compete off FB from deposited C3b preventing the formation of the C3 convertase. Furthermore, FH acts as a co-factor for factor I (FI) that can cleave C3b into inactive C3b (iC3b). Inactive C3b is unable to bind FB to form a new C3 convertase and cannot therefore stimulate a runaway amplification loop.

Consisting of both plasma and membrane bound proteins, the complement system is responsible for protecting the host from invading bacteria and other potential pathogens and can be activated by three separate pathways: the classical pathway, the lectin pathway and the alternative pathway [5]. During the activation of complement via the alternative pathway the protein C3b (an opsonin) becomes deposited on all local surfaces irrespective of whether the surface is host or foreign. It is then up to the host cell/tissue to deactivate the opsonin or risk amplifying the complement cascade leading to a local inflammatory response, recruitment of macrophages, the formation of the terminal membrane attack complex (MAC) and tissue damage (see Figure 2).

**Figure 3:**
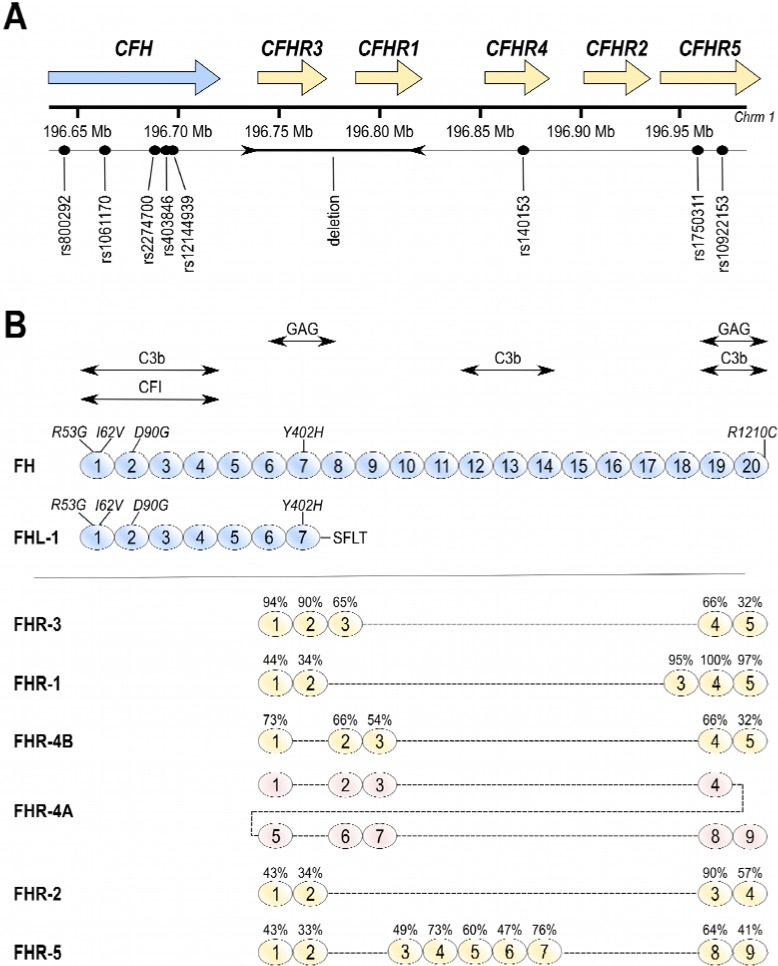
Complement factor H (*CFH*) and complement factor H related protein (*CFHR*) genes on chromosome 1 and the structures of FH, FHL-1 and FHR proteins. (**A**) The *CFH* and *CFHR* genes all occupy one stretch of chromosome 1 (1q32) known as the regulators of complement activation (RCA) cluster. The positions of some of the SNPs associated with AMD are shown; (**B**) A schematic diagram showing the twenty CCP domains of FH and the location of various ligand binding sites relevant to complement regulation. FHL-1, arising from a splice variation of the *CFH* gene, is identical to the first seven CCP domains before terminating with a unique *C*-terminal amino acid sequence of SFTL. The positions of coding variants in FH and FHL-1 associated with AMD are shown. FHR proteins 1–5 each share varying degrees of sequence homology to the FH protein, as indicated by a percentage above each domain. The identified alternative splice variant of FHR-4, known as FHR-4A, is also shown although it’s exact sequence and homology is still under investigation.

FH is a blood borne glycoprotein that is also produced locally by the RPE, and functions as a deactivator of the complement system. Although it can act in the fluid phase its main role is to bind to host surfaces and protect them against complement activation. FH consists of 20 complement control protein (CCP) domains and there is a splice variant of FH called factor H-like protein 1 (FHL-1) that contains the first 7 CCPs of FH and then a unique 4 amino acid carboxy-terminal tail (Figure 3B). FH (and FHL-1) acts primarily on the alternative complement pathway. It can disassociate factor B (FB; sometimes referred to as BbBa) from C3b on host surfaces thereby preventing the amplification of C3b deposition (see Figure 2C). In addition, it acts as a cofactor for FI. FI inactivates C3b by cleaving it to form inactive C3b (iC3b), but this requires the cofactor activity of FH or cell-surface bound complement regulators including complement receptor 1 (CR1) or the membrane cofactor protein (CD46). While both CR1 and CD46 play perhaps the more important role in protecting host cells from inappropriate complement attack, FH is the only regulator of complement to protect the extracellular matrix (ECM) [6]. During runaway complement activation two anaphylatoxins are released, C3a and C5a (see Figure 2A,B). These small proteins have a number of important effects including mediating chemotaxis, inflammation, and generation of cytotoxic oxygen radicals, along with inducing smooth muscle contraction, histamine release from mast cells, and enhanced vascular permeability [7].

The importance of FH as a complement regulator and conveyor of host protection is highlighted in cases where its function is perturbed leading to a number of non-ocular diseases, for example in the kidney conditions including atypical hemolytic uremic syndrome (aHUS) and Membranoproliferative Glomerulonephritis Type II (MPGNII—also referred to as dense deposit disease). In aHUS, mutations in the CCP19-20 region of FH are associated with an increase in complement turnover that subsequently leads to mesangial cell and matrix proliferation, arterial lesions and glomerular complement deposition [8]. Like AMD, MPGNII is a disease of the ECM, where defects in the regulation of the alternative pathway of complement, either by genetic alterations in genes including *CFH*, or the presence of auto-antibodies against FH, lead to deposits in the basement membranes of the glomeruli [9]. Intriguingly, sufferers of MPGNII can develop ocular drusen which are similar to those seen in AMD [9,10]. Poor regulation of the alternative pathway of complement by FH is also associated with Alzheimer’s disease [11], where changes in the sugar chain composition of the amyloid-β plaques associated with the disease [12] are believed to result in inadequate FH binding.

## 3. Factor H-Related Proteins

The complement factor H gene (*CFH*) is on chromosome 1 (1q32) and there are 5 genes (*CFHR1-5*) which encode complement factor H-related proteins (FHR1-5) that lie just downstream of the *CFH* gene (see Figure 3A). This region on chromosome 1 is known as the regulators of complement activation (RCA) cluster. The FHR proteins are also composed of CCP domains and these bear varying degrees of homology to the CCP domains found in FH (Figure 3B). Whilst the biological functions of these proteins have not been widely investigated there is evidence that they compete with FH by binding to C3b and/or glycosaminoglycans (GAGs), and that FHR-4 can form a novel C3 convertase [13]. As such they are now considered to be positive regulators of the alternative pathway of complement [14,15,16,17]. Recent studies have demonstrated that FHR-1, -2 and -5 circulate in the blood as dimers or higher oligomers [16,17], and a splice variant of the FHR-4 gene (now called FHR-4B) has been identified that results in a larger protein containing some repeating sequence (FHR-4A) [18]. As such, it is likely that any competitive function they may possess would be accentuated by oligomerization and repeating CCP domains resulting in higher avidity. These biochemical studies have assumed that the FHR proteins compete with FH, but in Bruch’s membrane (as discussed later) the main regulator is FHL-1 and they may compete differently with this truncated variant of FH [19].

## 4. Genetics of AMD and Functional Implications

Over the last decade the importance of genetics in determining AMD risk has become apparent. Two major loci have been identified. One is on chromosome 10 (10q31) near the *ARMS2/HTRA1* genes [3] and despite much work on this locus it remains unclear as to which of these genes is implicated and the mechanism whereby genetic variants modify risk. The other major locus is on chromosome 1 (1q32) involving *CFH* and *CFHR1-5* [3,4] in the RCA cluster (see Figure 3). Genetic alterations in the genes encoding other components of the complement cascade are also associated with AMD risk including FB/C2, C3, FI and C9, thereby providing strong evidence that this pathway is involved in AMD pathogenesis [3,20,21].

The locus on chromosome 1 is complex with multiple haplotypes having been identified that modify AMD risk [22]. Some non-coding variants in the *CFH* gene modify risk and this may be due to altered protein levels. For example, the protective rs6677604 allele is associated with raised plasma FH concentrations [23]. By modifying the protein sequence, coding variants could alter function; however, it is also possible that the alterations in protein sequence do not alter function and that the genetic markers are indicators of another mechanism whereby AMD risk is altered.

The genetic variant that was first identified as being associated with AMD and which remains one of the most important, is the rs1061170 coding variant in the *CFH* gene. This results in a Y402H amino acid substitution in the FH protein [24,25,26,27], or at position 384 when using the mature protein sequence numbering [28] (see Figure 3A). This polymorphism is present in ~35% of individuals of European descent [29] and results in a tyrosine residue being replaced by a histidine residue in the seventh CCP domain in FH (Figure 3B) [28]. Meta-analysis of 26 separate studies has demonstrated that individuals who are 402H heterozygous have a 2.3-fold increased risk of developing AMD, and 402H homozygotes have a 5.2-fold increased risk [29]. The potential consequences of the Y402H polymorphism are discussed in more detail below. Other coding variants in *CFH* include a relatively common I62V polymorphism, which is contained within a protective haplotype; this may have functional consequences as it is within the binding site for C3b [27].

More recently, less common mutations have been identified that are strongly associated with AMD (reviewed in [4]). A mutation that results in a R1210C amino acid substitution in FH confers high risk of AMD and is associated with early onset disease [22]. This mutation is also associated with aHUS. The mutation results in FH cross-linking to albumin via a disulphide bridge [30]. In addition, studies with a recombinant construct containing CCPs 8–20 suggest that the mutation affects GAG binding to cell surfaces [31,32]. Two other mutations have also been identified, R53C and D90G [33], where R53C decreased the ability of FH to dissociate FB from C3b, and both R53C and D90G showed decreased cofactor activity for the FI-mediated cleavage of C3b. The R53C mutation is likely to result in misfolding of the first CCP domain of FH, as it will disrupt the inherent disulphide bond conformation [34]; this domain is required for FI and C3b binding.

A number of the genetic alterations that modify AMD risk extend across the genetic region containing the 5 *FHR* genes, but it is currently unclear whether these affect the functions or levels of the FHR proteins. There is a common deletion of FHR-1 and -3 genes that is protective against AMD [14,35,36]; however this is not independent of intronic *CFH* SNP rs6677604 that is associated with increased plasma FH levels, but not with decreased FHR-1 levels [23].

## 5. Tissue Interactions of FH and FHL-1

Structurally, the Y402H polymorphism in FH occurs in the seventh CCP domain (see Figure 3B) but doesn’t perturb the overall conformation of the protein [37]. Furthermore, the rs106170 SNP which encodes the Y402H variant, is not associated with altered blood levels of FH or fluid phase complement activation [23,38]. It does, however, change the binding characteristics of FH to a number of ligands (reviewed in [6]) including *C*-reactive protein [39], Streptococcus M protein [40] and sulphated polyanions such as the glycosaminoglycan (GAG) chains [41] of proteoglycans. Binding to GAGs is the main mechanism by which FH anchors to ECM in order to protect it from inappropriate complement activation [42]. FH has two GAG binding sites, in CCP7 and CCPs19–20 (see Figure 3B). The AMD-associated Y402H polymorphism occurs in CCP7 and it has been shown to cause reduced binding of FH to human Bruch’s membrane and the inner choroid [43], where the main GAG classes involved in binding are heparan sulphate (HS) and dermatan sulphate. Decreased GAG binding could result in failure of localization of FH to these tissues and increased complement activation. However, whilst FH localizes to the surfaces of structures including Bruch’s membrane and drusen, the major complement regulator within these structures is FHL-1 [19]. FHL-1 retains all the same regulatory function of FH as it can bind C3b, FI and localize to tissues using its GAG binding site in CCP7. FHL-1 binds to Bruch’s membrane largely through interactions with HS, but it is unclear how FHL-1 interacts with drusen as this did not appear to be mediated by HS [19]. The FHL-1 in Bruch’s membrane could be derived from blood and/or synthesized by the RPE and it appears to be the predominant complement regular within Bruch’s membrane and drusen, because it can diffuse into these structures, whereas FH is too large to enter them [19].

This discovery may explain why the CCP7 GAG binding site interacts with HS in Bruch’s membrane in the eye, but does not bind HS in glomerular basement membranes in human kidneys [44]. Conversely, the CCP19–20 region of FH is unable to anchor FH to Bruch’s membrane in the eye, but can localize FH to HS in renal glomeruli [44]. This also explains the observation that a swathe of mutations in CCP20 of FH are associated with kidney diseases but, apart from the R1210C substitution, are not associated with an ocular phenotype [45,46]. In the case of FHL-1, a single amino acid change in its only Bruch’s anchoring site could make a difference to its binding [19]. The fact that the smaller, functionally active, FHL-1 protein can potentially diffuse through Bruch’s from the choroidal blood stream, whereas the much larger and glycosylated FH cannot, suggests that FHL-1 may have an important role in ECM protection throughout the body [19]. Similarly, FHL-1 has been detected inside drusen, while FH has been shown to coat the periphery of the druse [19]. This raises the possibility that the surface coating of full-length FH is protecting drusen from the natural immune clearance pathways of the complement cascade.

## 6. Effects of Aging on Ocular Immunity

Despite certain genes strongly influencing risk of developing AMD, it is an ageing disease that affects people over the age of 50 years. Age-dependent changes in the levels of metal ions [47] and products of oxidative stress [48,49] have been suggested as possible contributors to this phenomenon. However, from the perspective of complement regulation, a recent study has demonstrated an age-related change in the amount of HS GAG present in Bruch’s membrane with a decrease of 50% between the individuals in their 30’s *versus* their 80’s [50]. It was known that the expression of HS was remarkably varied in different layers of the eye [51,52] and that sulphation patterns and levels of HS can alter with age [53], but it had not been demonstrated previously that there is a marked loss of HS at this key site in AMD pathogenesis. Reduced levels of HS in Bruch’s membrane with age would result in less available binding sites for FHL-1 (or FH) to anchor to Bruch’s membrane. This could then be compounded by the Y402H polymorphism, as the 402H form of FHL-1 and FH binds HS in Bruch’s membrane poorly compared to the 402Y form due to its increased specificity for highly sulphated HS [19,41]. This combination of factors could explain the combination of genetic predisposition and ageing.

A number of questions remain regarding the biochemical mechanisms underlying the age-related loss of HS from Bruch’s membrane. HS is always attached to a core protein forming a proteoglycan (PG) [54] and it appears as though the loss of HS is due to a loss of the whole proteoglycan rather than enzymatic cleavage of the HS GAG chain from its core protein. Work is currently ongoing to identify which core proteins decrease with age, as preventing this loss of HS could provide a therapeutic strategy for preventing AMD.

## 7. Complement Based Therapies for AMD

Based upon the overwhelming evidence for the involvement of over-activation of the complement system in AMD, it is unsurprising that the cascade has become a therapeutic target. Some of the complement inhibitors that have been in clinical trials for AMD are discussed below.

A cyclic peptide inhibitor of C3 called POT-4 (Alcon, Hünenberg, Switzerland), delivered by intravitreal injection, has undergone a phase I clinical trial for neovascular AMD (ClinicalTrials.gov Identifier: NCT00473928). The results of this study have not been formerly published, but in a publication by Ambati *et al.* [55] a quote is included stating that “93% of patients showed no improvement in visual acuity”.

Eculizumab is a humanized monoclonal antibody against the complement protein C5 that reduces the release of C5a (a potent anaphylatoxin), dampens any inflammatory response and prevents the formation of the membrane attack complex (see Figure 2B). Originally designed for other complement driven diseases, such as aHUS, it has been investigated as a treatment for geographic atrophy in AMD. Disappointingly, Eculizumab did not affect the rate of progression of geographic atrophy [56]. One potential weakness of this study was that the participants were not genotyped to select patients who had strong genetic evidence of complement-mediated AMD. Another monoclonal antibody raised against C5, LFG316 (Novartis, Basel, Switzerland) is in clinical trials for geographic atrophy (NCT01527500, Phase II currently recruiting) and neovascular AMD (NCT01535950, phase II completed, but no results posted). With such strong parallels to the disappointing Eculizumab study described above it is uncertain whether LFG316 will show efficacy. Zimura (ARC1905, Ophthotech, New York, NY, USA) is an aptamer-based C5 inhibitor that blocks the cleavage of C5 into C5a and C5b fragments. It is delivered by intravitreal injection and is undergoing early-stage clinical trials for geographic atrophy and neovascular AMD (NCT00950638 and NCT00709527).

Lampalizumab (FCD4514S, Genentech/Roche, Basel, Switzerland) is a Fab antibody fragment that, unlike the therapeutics described above, specifically targets the alternative pathway of complement activation, and does not affect the classical or lectin pathways. This is achieved by binding factor D (FD), an enzyme that is plays a central role in activating the alternative pathway (see Figure 2A). This specific targeting of only one part of the complement cascade leaves the rest of the innate immune system intact to protect against pathogens. Phase II trials have been completed delivering Lampalizumab by intravitreal injection for geographic atrophy. Whilst the results have not yet been published, Roche has indicated that efficacy is seen and a phase III trial is commencing.

Finally, supplementation with recombinant FH (or proteins representing the functional domains of FH) has been proposed as a therapeutic strategy for AMD. The best mode of delivery remains uncertain. However, the recent finding that FHL-1 can traverse Bruch’s membrane from the choroid may provide hope for a systemic approach [19].

## 8. Conclusions

There is clear evidence that genetic variations at the RCA cluster at Chromosome 1q32 are strongly associated with AMD risk. At a protein level these alter the amounts, or by modifying sequence, the functions of FH, FHL-1 and possibly the FHR proteins, which act together to regulate the alternative pathway of the complement cascade. Dysregulation of complement in and around Bruch’s membrane brought about by insufficient binding of the regulators FHL-1 and FH and potentially compounded by an age-related loss in HS will lead to increased complement turnover, release of anaphylatoxins and a chronic local inflammatory response that may eventually result in visual loss from AMD. Insights into the mechanisms of AMD pathogenesis will allow new therapeutic strategies to be developed to prevent or treat this important cause of blindness.
